# CRISPR/Cas9-mediated mutagenesis of *FT*/*TFL1* in petunia improves plant architecture and early flowering

**DOI:** 10.1007/s11103-024-01454-9

**Published:** 2024-06-06

**Authors:** Mohamed Farah Abdulla, Karam Mostafa, Musa Kavas

**Affiliations:** 1 Faculty of Agriculture, Department of Agricultural Biotechnology, Ondokuz Mayis University, Samsun, 55200 Turkey; 2 The Central Laboratory for Date Palm Research and Development, Agricultural Research Center (ARC), Giza, 12619 Egypt

**Keywords:** *Petunia x hybrida*, *PhFT*, *PhTFL1*, Genome editing, CRISPR/Cas9, Compact plants

## Abstract

Petunias are renowned ornamental species widely cultivated as pot plants for their aesthetic appeal both indoors and outdoors. The preference for pot plants depends on their compact growth habit and abundant flowering. While genome editing has gained significant popularity in many crop plants in addressing growth and development and abiotic and biotic stress factors, relatively less emphasis has been placed on its application in ornamental plant species. Genome editing in ornamental plants opens up possibilities for enhancing their aesthetic qualities, offering innovative opportunities for manipulating plant architecture and visual appeal through precise genetic modifications. In this study, we aimed to optimize the procedure for an efficient genome editing system in petunia plants using the highly efficient multiplexed CRISPR/Cas9 system. Specifically, we targeted a total of six genes in *Petunia* which are associated with plant architecture traits, two paralogous of *FLOWERING LOCUS T (PhFT)* and four *TERMINAL FLOWER-LIKE1 (PhTFL1)* paralogous genes separately in two constructs. We successfully induced homogeneous and heterogeneous indels in the targeted genes through precise genome editing, resulting in significant phenotypic alterations in petunia. Notably, the plants harboring edited *PhTFL1 and PhFT* exhibited a conspicuously early flowering time in comparison to the wild-type counterparts. Furthermore, mutants with alterations in the *PhTFL1* demonstrated shorter internodes than wild-type, likely by downregulating the gibberellic acid pathway genes *PhGAI*, creating a more compact and aesthetically appealing phenotype. This study represents the first successful endeavor to produce compact petunia plants with increased flower abundance through genome editing. Our approach holds immense promise to improve economically important potting plants like petunia and serve as a potential foundation for further improvements in similar ornamental plant species.

**Supplementary Information:**

The online version contains supplementary material available at 10.1007/s11103-024-01454-9.

## Key message

A CRISPR-mediated genome editing in Petunia results in compact, early-flowering mutants, paving new pathways in the advancement of ornamental plant breeding.

## Introduction

Ornamental plants, also known as decorative or flowering plants, are economically important and cultivated worldwide for their aesthetic appeal and decorative qualities. Ornamental plants have a rich and diverse history that spans thousands of years, intertwined with human civilization and culture (Ching et al. 2017). Today, ornamental plants continue to be cultivated and appreciated for their aesthetic value, environmental benefits, and contribution to human well-being. They are used in private gardens, public spaces, landscaping projects, and interior design, adding beauty, color, and texture to our surroundings (Hale et al. 2011). In the transition from the 20th to the 21st century, many ornamental plants have become more accessible and diverse than ever before for human consumption (Abdulla and Çelikel 2019).

Petunia (*Petunia x hybrida*) is a popular ornamental bedding plant and is widely grown throughout the world. Petunia is also considered a model crop to investigate the functional roles genes in ornamental plants (Liang et al. 2014). Unlike fruits and vegetables, which are mainly grown for human consumption, ornamental plants are grown for their aesthetic value. In the realm of ornamental bedding plants, consumers lean toward smaller, more compact flower plants that concurrently maintain high-quality attributes. Particularly, the advantages of smaller plants lie in their space-efficient cultivation, ease of handling, and reduced shipping costs (Suh et al. 2020). A common commercial treatment for growth regulation in ornamental crops is the application of synthetic growth retardants, which mostly interfere with gibberellin (GA) biosynthesis (Demir and Çelikel 2018; Sajjad et al. 2017). These compounds effectively reduce the length of the stem, but the treatments, unfortunately, also result in delayed flowering, which is an undesirable side effect for producers. The principal concern associated with the application of synthetic growth retardants lies in their possible negative influence on both the environment and human health. Researchers and politicians have extensively investigated and discussed this particular aspect (Rademacher 2000).

Transgenic technologies enhance ornamental plants by modifying or engineering changes in the plants’ genomes. For example, genetic and biochemical analysis of floral pigments has led to the creation of blue-hued carnations, roses (Kishi-Kaboshi et al. 2018; TANAKA et al. 2010), and chrysanthemums (Noda et al. 2017). Since 2013, an efficient genome-editing tool has been developed. This tool is based on the bacterial Clustered Regularly Interspaced Short Palindromic Repeats (CRISPR)/ CRISPR-associated protein 9 (Cas9) system. This system is a constituent of the bacterial adaptive immune system and has been extensively used in plant genome-editing for the past decade (Cardi et al. 2023; Lee and Sashital 2022). CRISPR/Cas9 genome editing technology can be utilized to improve the plant architecture and modify the color, fragrance, size, and shelf life of the flowers. Genetic transformation of ornamental plants enables the production of high-quality flowers and generates plants with novel colors and architecture (Erpen-Dalla Corte et al. 2019; Noda et al. 2017). However, just like crop plants, certain ornamental cultivars pose challenges in terms of successful transformation using *Agrobacterium*. In these cases, a proof-of-concept study is typically required before employing strategies for the targeted editing of useful traits aimed at enhancing the cultivar (Sirohi et al. 2022).

*FLOWERING LOCUS* T (*FT)* and *TERMINAL FLOWERING-LIKE* 1 (*TFL1)* are widely available in plants. In *Arabidopsis*, their function is linked with the meristem and flowering regulation by repression of flowering and promoting vegetativeness (Baumann et al. 2015; Hanzawa et al. 2005). Both genes were found to contain a conserved phosphatidylethanolamine-binding proteins (PEBPs) domain. Expression of PEBP containing *FT* is said to be decreased on floral transition (Kinoshita and Richter 2020). Members of PEBP, including *FT*, *TWIN SISTER OF FT* (*TSF*), *TERMINAL FLOWER-LIKE1* (*TFL1*), *Arabidopsis ortholog of CENTRORADIALIS* (*ATC*), and *BROTHER OF FT*, are shown to form a complex network regulating flowering in plants (Collani et al. 2019; Kim et al. 2013; Périlleux et al. 2019). While *FT* and *TSF* promote flowering, *TFL1*, *ATC*, and *BROTHER OF FT* (*BFT*) counteract this process by inhibiting floral promoters or interfering with FT’s function and promoting vegetative development and branching (Lifschitz et al. 2014; Zhu et al. 2020). In Arabidopsis, the amino acids TY-85 and Gln-140 are the main features that could distinguish the functional characteristics of FT and the TFL protein (Tsukamoto et al. 2016). In petunia, five *FT* orthologous genes were reported, four in tomato, and only two orthologous in *Arabidopsis* (Cao et al. 2016; Wu et al. 2019). In the *TFL1-like* subfamily, six genes are reported in both petunia and tobacco, five in tomato, and three in *Arabidopsis* (Wang et al. 2015; Wu et al. 2019).

The main objective of this study was to optimize the development of transgenic petunia plants through CRISPR-mediated genome editing, utilizing *Agrobacterium tumefaciens* for the mutation of pivotal genes that regulate plant architecture. In addition, to engineer transgenic mutant petunia lines exhibiting early flowering, increased flower rate, and a compact, shortened fluorescence with reduced internodal lengths. To our knowledge, this study is the first of its kind to investigate and address these specific aspects of petunia plant modification, opening up new possibilities for enhancing petunia traits and offering potential applications in horticulture and ornamental breeding.

## Materials and methods

### Plant material and growth environment

In this study, *Petunia × hybrida* (*petunia*) cv. ‘Mitchell Diploid’ seeds were procured commercially. Following surface sterilization of seeds with 1 min 70% ethanol followed by 20 min washing with 20% bleach, seeds were germinated on half-strength Murashige and Skoog MS media. After six weeks, a 5 cm shoot explant with a single leaf was transferred to freshly prepared MS media to facilitate further growth. Approximately four weeks later, the transformation process was initiated when the leaves reached approximately 2 cm in diameter. Leaf disks measuring 1 cm^2^ were excised for transformation. For each construct approximately 40 petri dishes were allocated with five to seven leaf discs each. Explants were allowed to grow on growth champers with a constant temperature of 25 °C and supplemented with a bright white LED light (250 µmol m − 2 s − 1) for 16 h and 8 h darkness. Explants that successfully developed roots from tissue culture were transferred into potting soil, a mixture of peat moss and perlite, in a 2:1 ratio. The plants were relocated to an acclimatization room with high humidity (90%) for two weeks. After this period, the plants were placed in growth chambers set at a continuous temperature of 25 °C, with a long-day photoperiod (16 h of light and 8 h of darkness at 60% relative humidity). Supplementary LED lighting (250 µmol m − 2 s − 1) was provided. Manual irrigation was performed, adhering to a standard fertilization regimen using NPK plus trace elements and Hoagland solution (Secgin et al. 2021).

### Sequence retrieval of FT/TFL1, phylogenetic relationships, and protein-protein interaction network

The genomic DNA sequences of four paralogous genes of *TERMINAL FLOWER-LIKE 1* (*TFL1*) (*PhTFL1a*, Peaxi162Scf01281g00001.1; *PhTFL1b*, Peaxi162Scf00091g00096.1; *PhTFL1c*, Peaxi162Scf00040g02110.1; *PhTFL1d*, Peaxi162Scf00163g00521.1), two paralogous genes of the *FLOWERING LOCUS T* (*FT*) (*PhFT1*, Peaxi162Scf00254g00117.1; *PhFT2*, Peaxi162Scf00658g00029.1) were extracted from the *Petunia axillaris* draft genome sequence in the Sol Genome Network database (https://solgenomics.net/organism/Petunia_axillaris/genome) via Blastn using. The previously reported nucleotide sequences of the *Slsp* and *SlSP5g* (Kwon et al. 2020) were used as a query for the blast search in NCBI Blastn (https://blast.ncbi.nlm.nih.gov/Blast.cgi). Additionally, protein sequences corresponding to *FT/TFL1* in *Solanum lycopersicum*, *Oryza sativa*, and *Arabidopsis thaliana* were retrieved via the Blast search tool in Phytozome v13 using *AtFT* and *AtTFL1* as the query sequences. The total protein sequences were initially aligned with ClustalW in MEGA 11 software, utilizing default settings. Subsequently, the alignment data were uploaded to the IQ-TREE web tool to conduct phylogenetic relationship analysis using ModelFinder for the best fit model and ultrafast bootstrap (1000 replicates) (Hoang et al. 2017; Kalyaanamoorthy et al. 2017; Nguyen et al. 2014). The resulting phylogenetic tree was visualized using the online ITOL tool and edited using Adobe Illustrator. The STRING protein-protein interaction database version 12 was used to identify the interacting protein networks and functional annotations. Protein sequences of PhFT and PhTFL1 were used as queries against the *Arabidopsis* genome as a plant model with a minimum interaction score of 0.7 (Kavas et al. 2022; Szklarczyk et al. 2022). Protein sequences and Protein-protein interaction (PPI) networks can be accessed in supplementary Table 2.

The analysis of the motif, conserved domain, and the exon: intron structure of PhFT/PhTFL1 protein sequences was done utilizing the Generic Feature Format version 3 (GFF3) and the protein sequences downloaded from the SOL genomics database (Fernandez-Pozo et al. 2014). The motifs were predicted and analyzed by MEME-Suite search using the protein sequences of PhFT and PhTFL1 as the query. Each motif underwent individual scrutiny, with only those possessing an e-value of less than 1e-10 considered for motif detection to ensure precision. Conserved domain structures were extracted from NCBI CD search (Kavas et al. 2021, 2022). TBtools II software was employed for the analysis and visualization of this data (Chen et al. 2023).

### sgRNA design and construction of CRISPR/Cas9 plant expression vector

**Fig. 1:**
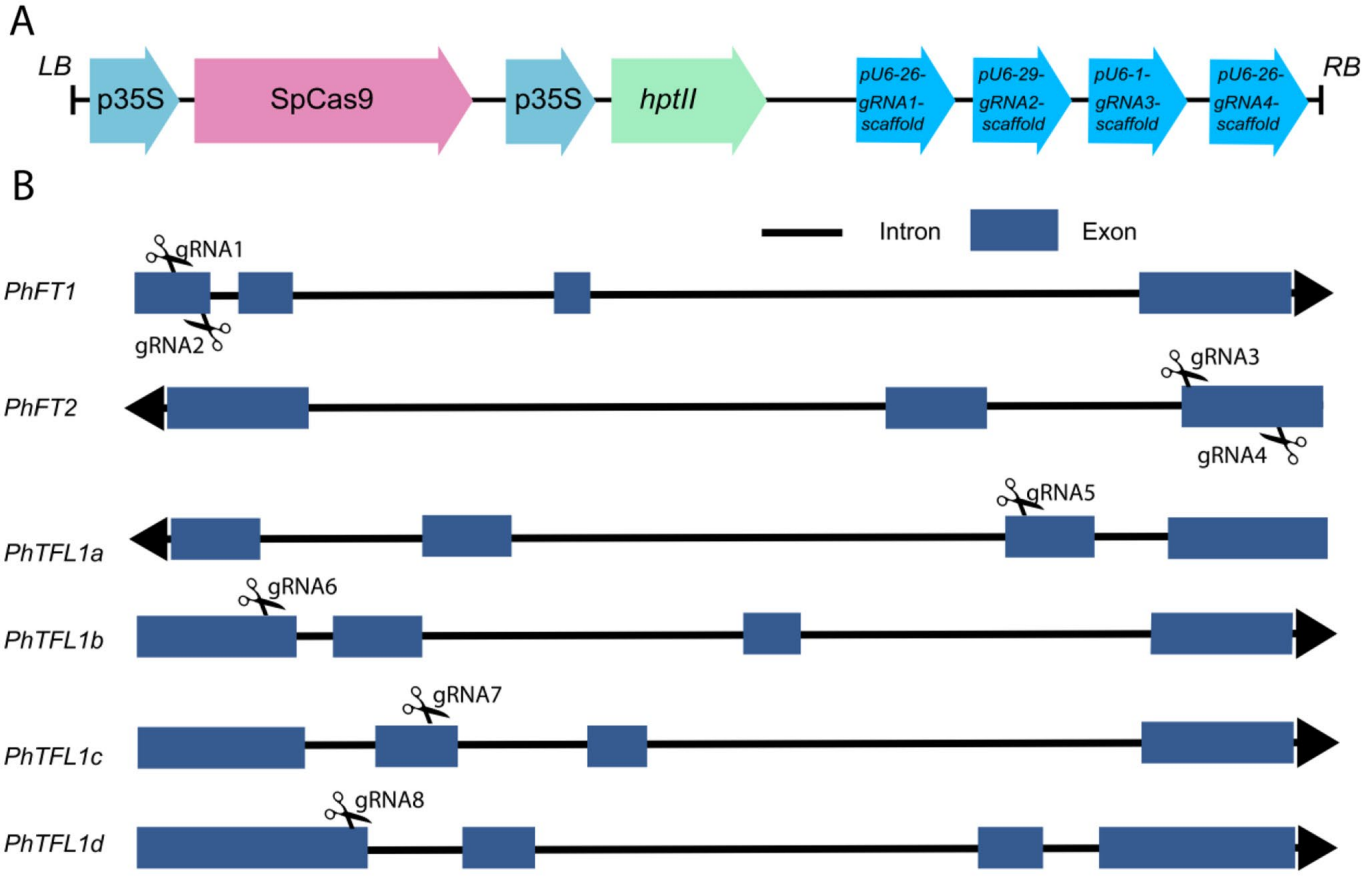
Graphical Representation of Plant Expression Design and sgRNA Structure. **A**. Illustration of the binary plant expression vector pHSE401 utilized in Agrobacterium-mediated transformation. The arrows indicate the directionality, with *‘hptII’* representing the hygromycin-resistant gene under the control of an enhanced cauliflower mosaic virus promoter (*CaMV 35S*). The *Streptococcus pyogenes Cas9* (*SpCas9*) is also controlled by *CaMV 35S*. The sgRNAs expression is controlled by *Arabidopsis Ubiquitin 6 (AtU6)* promoter variants alongside their respective terminators and RNA scaffold. **B**. Depiction of gene structure of targeted genes: Arrows indicate the transcript direction, black lines represent introns, and the rectangular blue boxes represent the exons. The sgRNA target sites are marked with scissors within the gene structure diagram

The CRISPR/Cas9-meadiated mutagenesis was conducted following the previously well-established methodologies (Gökdemir et al. 2022; Secgin et al. 2022; Xing et al. 2014) (Fig. 1). In brief, two sgRNAs (sgRNA1 and sgRNA2) targeting the first exon of *PhFT1*, and two sgRNAs (sgRNA3 and sgRNA4) targeting the first exon of *PhFT2* and four sgRNAs (sgRNA5, sgRNA6, sgRNA7, and sgRNA8) targeting each of the four *PhTFL1 (PhTFL1a, PhTFL1b, PhTFL1c, PhTFL1d)* paralogous. All the sgRNAs were designed using the online toolkits CRISPROR and CHOPCHOP simultaneously (Table 1).

**Table 1:** Efficiency and sequence of designed gRNA and their target sites

sgRNA No.	Target sequence	Target Gene ID	Genomic location	GC (%)	Self- comp	MM0	Efficiency
1	AAGGTCAACTAGCCTAAGAG	*PhFT1*	Peaxi162Scf00254:1101201	45	4	0	61.86
2	AGTGGTTTACAACACTAGGG	*PhFT1*	Peaxi162Scf00254:1101219	45	2	0	69.85
3	CGTTGATAACATCTCTACTT	*PhFT2*	Peaxi162Scf00658:248623	35	0	0	40.46
4	CTCCAATATCAACCCTAGGT	*PhFT2*	Peaxi162Scf00658:248569	45	1	0	44.5
5	TGAAGATGTTCCCGAAGGTA	*PhTFL1a*	Peaxi162Scf00091:954099	45	0	0	44.03
6	CCTCTGTCACTTCTAAACCT	*PhTFL1b*	Peaxi162Scf01281:65312	45	0	0	54.64
7	CTCTTAGATAAGGATCACTA	*PhTFL1c*	Peaxi162Scf00040:2158557	35	1	0	61.56
8	CCTCTGTCACTTCTAAACCT	*PhTFL1d*	Peaxi162Scf01281:65312	45	0	0	54.64

Self-Comp; Self complementary score, MM0; number of predicted mismatches at 0 bp

Subsequently, plant expression binary vectors carrying four gRNAs were constructed through the Golden Gate cloning system, which involved the digestion and ligation of three gRNA-expressing vectors with a plant expression backbone vector pHSE401, a gift from Qi-Jun Chen (Addgene plasmid #62,201; http://n2t.net/addgene:62201) (refer to Supplementary data 2).

## Agrobacterium-mediated transformation

Once the sgRNA sequences were confirmed with enzyme digestion and Sanger sequenced, the final plant expression vectors were introduced into the petunia leaves via *Agrobacterium tumefaciens* (strain *GV3101*)-mediated transformation. This process commenced by initially co-transfecting the leaf explants with the bacterial culture in MSG broth for 20 min. The excess bacterial broth was removed by gently placing the explants on sterile filter papers before placing them in co-cultivation media (see Table 2). Co-cultivation was carried out in a dark growth chamber at 25 °C for two days. Following this period, the explants were washed in liquid MS media supplemented with two consecutive antibiotics, timentin (320 mg/mL) and cefotaxime (250 mg/mL), for 15 min. Subsequently, the explants were placed on sterile filter papers to remove excess media. Later, the explants were placed on selective shoot induction 1 (SSI1) media until callus were formed and shoot initiation was visible. The initiated shoots were then subcultured into another selective media, SSI2, to allow further shoot growth. Once the explants produced shoots exceeding 2 cm, they were transferred to selective root-inducing (SRI) media.

**Table 2:** In-vitro media contents used in the tissue culture in petunia

	PC (mg/mL)	CC (mg/mL)	SSI1 (mg/mL)	SSI2 (mg/mL)	SRI (mg/mL)
BAP	2	2	2	1	-
NAA	0.5	0.5	0.5	0.1	0.5
Acetosyringone	20	20	-	-	-
Hygromycin	-	-	20	30	50
Timentin	-	-	200	200	200
Cefotaxime	-	-	125	125	125

BAP: 6-benzylaminopurine; NAA, 1-Naphthaleneacetic acid; PC, Pre-culture; CC, Co-culture; SSI, Selective shoot induction; SRI, Selective root induction

## Phenotyping and genotyping of transgenic lines

Genotyping of transgenic lines commenced with PCR amplification of *hptII* to confirm the successful integration of T-DNA into the plant genome. The total DNA was isolated manually using the CTAB DNA extraction method with modification (Schenk et al. 2023). Subsequently, Sanger sequencing was carried out on positive transgenic lines, using PCR-amplified gene fragments encompassing the gRNA-targeted site. For phenotyping the transgenic lines, all measurements were conducted manually using a digital caliper and a standard 30-cm ruler, particularly when at least half of the flowers on the wild-type line had opened. The length from the shoot’s bottom to its tip was measured in terms of plant height. The space between leaves was measured to determine internode length, and the average was recorded. Peduncle length was measured for each flower inflorescence, and the average of all measurements was calculated. The number of flowers was tallied as the total number in the inflorescence. Flowering time was recorded when the first flower was observed in the inflorescence. Each line comprised three individual replicates.

For the genotype analysis, the extracted DNA was used as a template for Polymerase Chain Reaction (PCR) amplification using High fidelity Q5 Polymerase (NEB, UK). Purified PCR products of mutated lines and WT lines were then sent for sequencing of the targeted region. Sanger sequencing was employed for the accurate analysis using gene-specific primers (Table S1). The Synthego ICE Analysis (https://ice.synthego.com) was employed to analyze the mutation type and the mutation rates of selected gRNAs. This comprehensive approach allowed for a detailed examination of the genetic alterations induced by the gRNA in the genome.

### RNA extraction and gene expression analysis by qRT-PCR

Green, healthy leaf tissues were excised from each line as samples, promptly frozen in liquid nitrogen, and stored at -80 °C until they were utilized for RNA isolation. RNA extraction was carried out manually using the CTAB method, following previously established protocols (Seçgin et al. 2020). The concentration and integrity of the RNA were assessed using the NanoDrop™ 2000/2000c spectrophotometer (Thermo, USA) and electrophoresis on a 1.5% (w/v) agarose gel. A consistent concentration of 1000 ng of RNA was employed to synthesize the first strand of cDNA. The cDNA synthesis was performed utilizing the iScript cDNA synthesis kit (Bio-Rad, USA), following the manufacturer’s protocol. For the qRT-PCR reaction, GoTaq® qPCR Master Mix (Promega, USA) was utilized, following the manufacturer’s instructions, and 1 µL of cDNA was added to a 20 µL reaction. qRT-PCR was conducted in triplicate on the Agilent Mx3000P (Agilent, USA) under the following conditions: 95 °C for 2 min, followed by 40 cycles of 95 °C for 15 s and 60 °C for 1 min. The 2^−∆∆CT^ method was applied using Excel software to compute the relative expression. *Petunia × hybrida Elongation Factor 1 α (PhEF1α)* served as the internal reference gene (Mallona et al. 2010), and wild-type plants were employed for normalization.

### Statistical analysis

For the quantitative analyses of the data significance, SPSS 26 software was employed (SPSS Inc., USA. The statistical assessments included a one-way analysis of variance (ANOVA) with Tukey’s post hoc test, as deemed appropriate for the analysis. All original data and the specific sample sizes for each experimental group can be referenced in Table S1.

## Results

### Identification and structural characterization of*PhFT/PhTFL1*

**Fig. 2:**
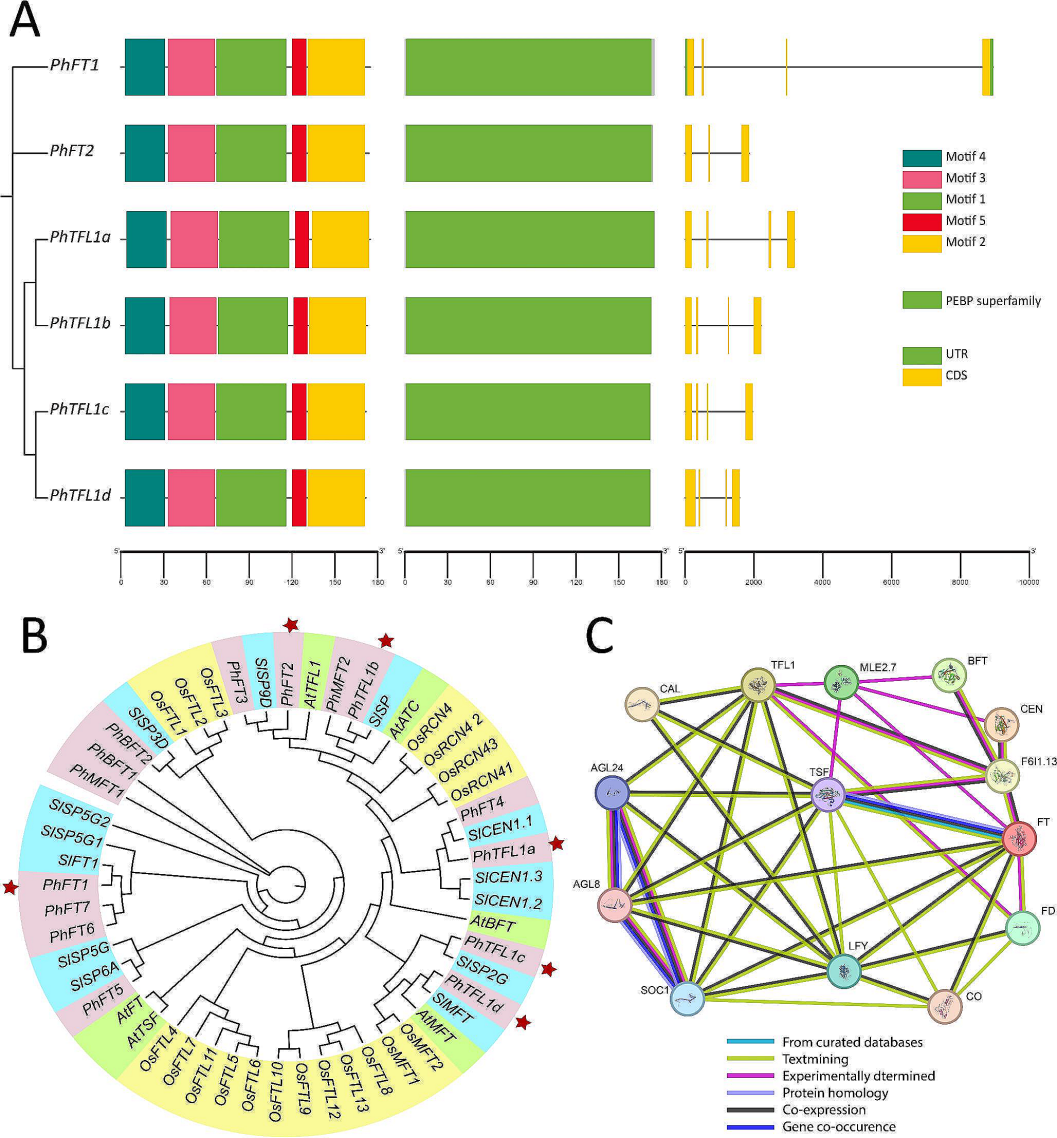
Phylogenetic tree of the *FT/TFL1* gene members, genetic structure, and interactions. **A**, Graphical representation of the genetic structure of studied genes. The graph was constructed using the TBTools software. The phylogenetic relationship was constructed using the QTREE online software using default settings. **B**, Phylogenetic relationship between FT/TFL1 protein sequences of selected angiosperms: *Solanum lycopersicum* (blue), *Petunia x hybrida* (purple), *Oryza sativa* (yellow), and *Arabidopsis* (green). A maximum likelihood phylogenetic tree was generated using the online IQ-tree web tool. The genes targeted for the CRISPR/Cas9-mediated mutagenesis are marked with red stars. **C**, Protein-Protein interaction (PPI) network between the FT/TFL1 and their related proteins predicted by the STRING database v12

A total of two homologous genes were selected from the blasted *Arabidopsis thaliana Flowering Locus* gene family and named *PhFT1* and *PhFT2*. A total of four homologous genes were selected to represent the *Terminal flower-like 1* (*PhTFL1a*, *PhTFL1b*, *PhTFL1c*, and *PhTFL1d*). A gene structure analysis was constructed, including a phylogenetic tree to show the relationship between the selected genes (Fig. 2A). Based on the genetic structure of the selected genes, they all consisted of the same motifs. In addition, all the targeted genes had an identical conserved domain, namely, PEBP (PhosphatidylEthanolamine-Binding Protein). The difference was seen between the genes in their genetic architecture. The genetic architecture showed that the number of exons was four for all genes except for *PhTF4*, which had only three exons.

To better analyze the sequence similarity and relationship between the FT/TFL1 proteins among angiosperms, protein sequences of FT/TFL1 from *Arabidopsis* were used as blast query to extract the homologous gene members from four distinct organisms: *Solanum lycopersicum* (13 genes), *Oryza sativa* (19 genes), *Arabidopsis* (6 genes), and *Petunia x hybrida* (15 genes). A total of 53 sequences were extracted, and alignment was performed using the ClustalW 2.0 program within the MEGA11 software. Then, using the generated alignment file, a maximum likelihood phylogenetic tree was generated with the best-fit model (JTT + G4) using the IQ-TREE web tool (Fig. 2B). The analysis revealed that the Petunia FT/TFL1 proteins have closer relationships with those from *Solanum lycopersicum*, given that they belong to the same *Solanaceae* family. Conversely, the FT/TFL1 proteins from rice displayed higher conservation levels and did not exhibit a close relationship with proteins from other organisms.

The PPI network analysis was used to estimate the interactions between the Petunia FT/TFL1 proteins and their related proteins, using STRING v12 and the *Arabidopsis* as a model plant. The analyses indicated that FT and TFL1 are closely related and exhibit strong interactions with florigenic proteins such as FD, AGL, BFT, CEN, LFY, MLE, CO, and SOC (Fig. 2C, supplementary data 1).

### Generation of stable genome-edited petunia plants

**Fig. 3:**
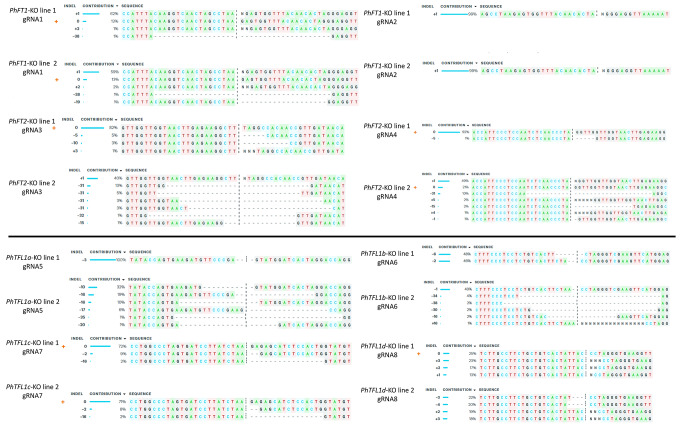
Illustrations show the sequence analysis of the target region in the T0 mutant lines compared to the wild-type sequence using the online SYNTHEGO ICE tool. Vertical black dots represent the Cas9 cleavage site, while the black hyphen represents deletions. The wild-type sequence is marked by a “+” symbol on the left

As presented in Fig. 1; Table 1, eight specific sgRNAs were designed to target the CDS regions of *PhFT1, PhFT2*, *PhTFL1a*, *PhTFL1b*, *PhTFL1c*, and *PhTFL1d* based on their highest efficiency rates. From each construct (*PhFT*-KO Line 1, *PhFT*-KO Line 2, *PhTFL1*-KO Line 1, and *PhTFL1*-KO Line 2), two T0 transgenic genome-edited lines were selected, and the details of the editing nature at all target sites are illustrated in Fig. 3. Initially, PCR amplification was conducted to confirm the transgenic nature of the lines using the *hptII* gene-specific primers. Subsequently, the targeted regions of transgenic lines and wild-type fragments within the CDS were PCR amplified and sent for Sanger sequencing. The resulting sequences were analyzed against the wild type sequence using the online ICE tool. Various mutation types induced by the sgRNAs were observed across all transgenic lines, including bi-allelic, multi-allelic, and mono-allelic mutations.

### Knock-out of *PhFT/PhTFL1* reduces plant height and enhances early flowering

**Fig. 4:**
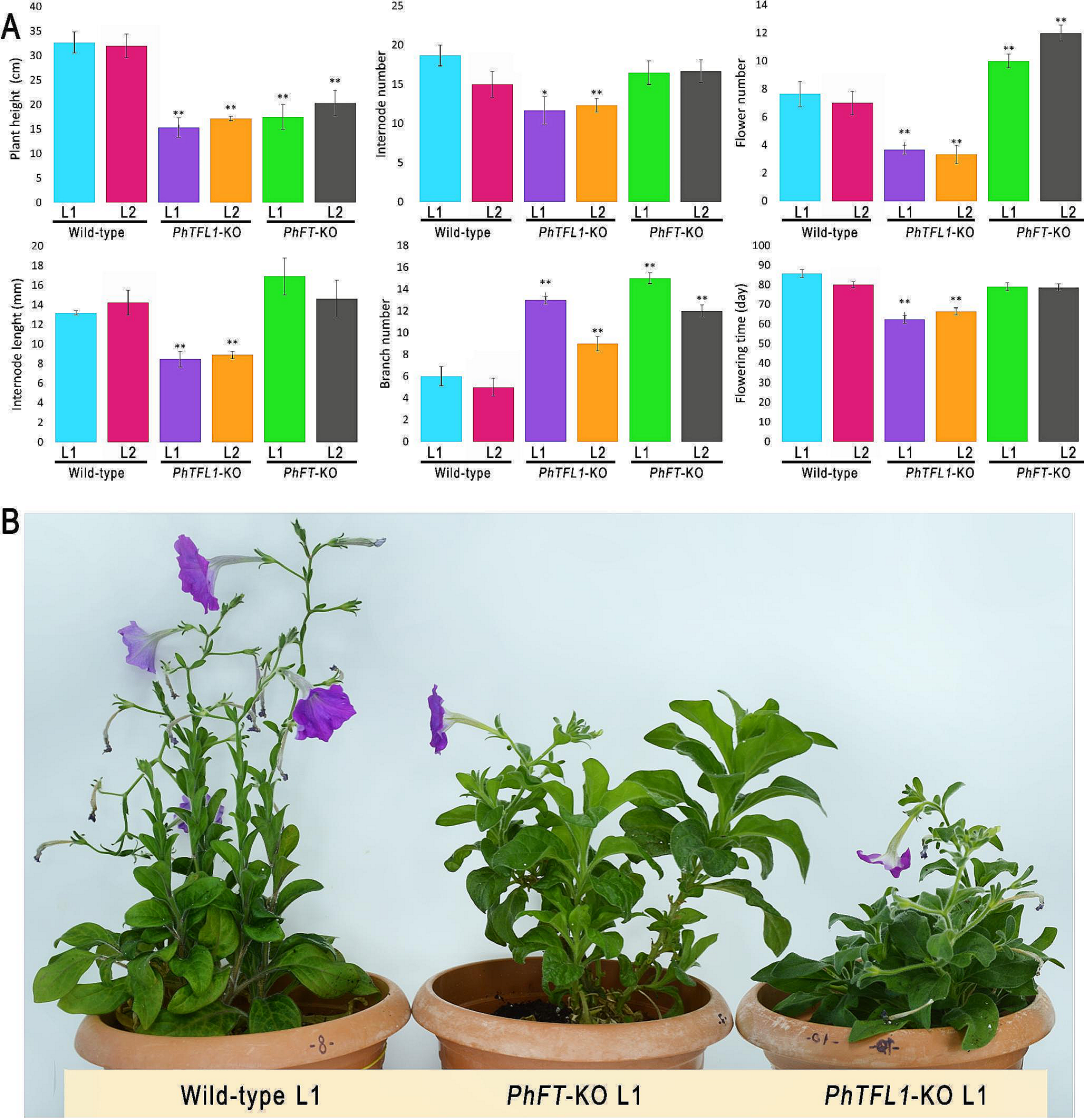
Phenotypic analysis comparison between mutant (*PhTFL1*-KO and *PhFT*-KO) and Wild-type Lines. **(A)** Quantification of Plant height (shoot length), number of internodes, flowers per inflorescence, internode length, number of branches, and time to first flower in mutant and wild-type lines. Data represent means from three replicates with error bars denoting standard errors. Statistical significance indicated by ANOVA Test (*P < 0.05, **P < 0.001). **(B)** Comparative representation of wild-type plants against the *PhTFL1*-KO and *PhFT*-KO CRISPR/Cas9-mediated mutant lines

After the successful generation of the *PhFT*-KO and *PhTFL1*-KO mutant petunia lines, some physiological analysis was conducted to assess the effect of the mutation on the plant’s architecture (Fig. 4A, B). Firstly, we observed a significant decrease in plant height in the mutant lines compared to the wild-type lines. At the same growth stage, the *PhTFL1*-KO mutant lines exhibited the shortest height, with an average of 16.25 cm. In contrast, the *PhFT*-KO mutated lines showed an average plant height of 18.92 cm, while the wild-type lines displayed an average height of 32.33 cm. This difference in height indicates a reduction of 49.74% between the wild-type and the *PhTFL1*-KO mutant lines. Similarly, a reduction of 41.49% between the *PhFT*-KO mutant lines and the wild-type lines. These findings highlight the impact of the *PhTFL1*-KO and *PhFT*-KO mutations on plant height, underscoring the crucial role these genes play in regulating plant growth.

A significant decrease in the number of internodes was observed between the mutant and wild-type lines. In *PhTFL1*-KO lines, a significant reduction in internode count was observed when compared to the wild-type lines. While the wild-type exhibited an average of 16.8 internodes, the *PhTFL1*-KO lines displayed an average of 12 internodes per fluorescence. Interestingly, the *PhFT*-KO lines did not show a significant change in internode count when compared to the wild-type counterparts (Fig. 4A).

There was a statistically significant difference in the number of flowers per inflorescence between the wild-type and mutant lines. In contrast to the wild-type lines, which had an average of 7.3 flowers per inflorescence, the *PhTFL1*-KO lines exhibited the lowest number, averaging 3.5 flowers. Surprisingly, the *PhTFL1*-KO lines displayed a higher number of flowers, averaging 11 flowers per inflorescence. These findings underscore the substantial impact of the *PhTFL1* and *PhFT* mutation on the number of flowers (Fig. 4A).

A significant effect on internode length per inflorescence was observed comparing wild-type to mutant lines. The *PhTFL1*-KO lines showed the shortest internode length, with an average of 8.5 cm in line 1 and 8.9 cm in line 2. In contrast, the wild-type lines displayed longer internodes, with an average of 13.18 cm in line 1 and 15.5 cm in line 2. Conversely, no statistically significant difference in internode length per inflorescence was observed between the wild-type lines and the *PhFT*-KO lines. These findings highlight the significant role of the *PhTFL1* gene in regulating internode length and, hence, the development of a more compact plant architecture (Fig. 4A).

To assess the impact of the mutations on the branching habit of the petunia plants, the primary branch numbers were counted for each plant. A statistically significant difference was recorded between the mutant and wild-type counterparts. The *PhTFL1*-KO lines showed an increase in the primary branches, averaging 11 per plant. On the other hand, the *PhFT*-KO lines showed an average of 13.5 branches. In contrast, the wild-type lines averaged 5.5 branches per plant. Consequently, the mutant lines developed a compact, bushy appearance due to the increased branching observed compared to the wild-type, indicating the substantial influence of these mutations on the overall plant architecture (Fig. 4A, B).

The time to first flower was also recorded for each plant. A significant increase in early flowering time was observed in the mutant lines of *PhTF1*-KO compared to their wild-type counterparts. The *PhTFL1*-KO mutant lines (line 1 and line 2) exhibited the earliest flowering, displaying a remarkable reduction in the time to flowering with 18.5 days on average compared to the wild-type lines. The *PhFT*-KO mutant lines also recorded significant early flowering time with an average of four days compared to the wild-type lines. These findings highlight the role of both *PhFT*-KO and *PhTFL1*-KO in significantly inducing early flowering in petunia plants (Fig. 4A, B).

### Effect of CRISPR/Cas9-mediated mutation of *PhFT/PhTFL1* on gibberellic acid pathway genes and interacting genes

**Fig. 5:**
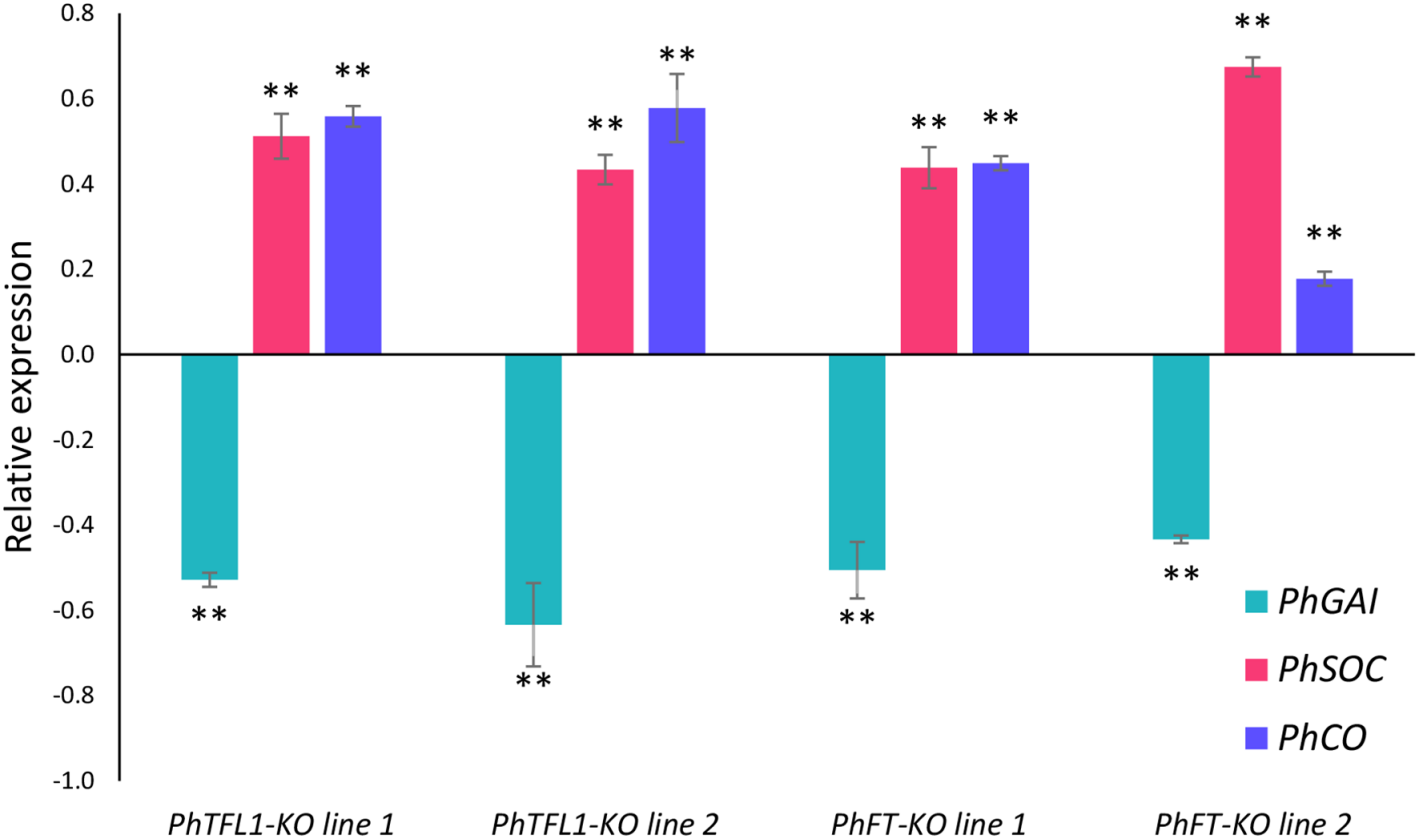
Expression Analysis of *PhGAI*, *PhSOC*, and *PhCO* in CRISPR/Cas9-mediated *PhTFL1*-KO and *PhFT*-KO Using qRT-PCR. Data depict mean LOG values from three replicates, with error bars representing standard errors. Statistical significance was determined using the ANOVA Test (*P < 0.05, **P < 0.001)

Gene expression analysis was conducted to examine how the *PhTFL1* and *PhFT* mutations influence the activity of related genes. Specifically, a GRAS family transcription factor protein, *GIBBERELLIC ACID INSENSITIVE* (*PhGAI*), was chosen to assess their expression levels in mutant versus wild-type plants using Real-Time Quantitative Reverse Transcription PCR (qRT-PCR). *PhGAI* exhibited downregulation in both *PhFT*-KO and *PhTFL1*-KO lines, indicating a significant regulatory connection between *PhFT*, *PhTFL1*, and *PhGAI* (Fig. 5).

Furthermore, the expression level of the MADS-Box family *SUPPRESSOR OF OVEREXPRESSION OF CONSTANS* (*PhSOC*) was analyzed. Results showed a significant increase in the expression level of *PhSOC* transcripts across all *PhFT* and *PhTFL1* mutant lines compared to the wild type, suggesting that *PhFT* and *PhTFL1* negatively regulate the transcription of *PhSOC*. Finally, the expression analysis of Zinc finger protein *CONSTANS-LIKE* (*PhCO*) had a similar expression pattern as the *PhSOC*, displaying an upregulation profile in mutant lines in comparison to the wild type lines.

## Discussion

Various types of synthetic plant growth regulators (PGRs) are commonly employed in the production of compact flowering pot plants. However, many of these chemicals pose potential risks to the environment and human health (Sørensen and Danielsen 2006). Smaller plant sizes generally enhance the quality of flowers or bedding plants, subsequently reducing greenhouse space requirements, hedge trimming costs, and the use of synthetic growth inhibitors for ornamental plants (Rademacher 2015). Chemicals that result in retarded growth also inhibit the production of gibberellic acid (GA) (Rademacher 2000). However, it is important to note that GA also plays a crucial role in controlling plant height, flowering time, and overall morphogenesis of plants (Fleet and Sun 2005). Therefore, different approaches have been taken, including genetic manipulation (Mekapogu et al. 2023).

In this study, two orthologs of FT (*PhFT1* and *PhFT2*) and four orthologs of *TFL1* (*PhTFL1a*, *PhTFL1b*, *PhTFL1c*, and *PhTFL1d*) were targeted. The resulting mutant lines exhibited significant changes in plant architecture. CRISPR/Cas9-mediated mutagenesis of *PhTFL1* and *PhFT* led to petunia plants with shorter shoot lengths, multiple branching, and an aesthetically appealing look. Similar outcomes were observed when targeting the *TFL1* genes in other mutated plants, such as tomato, *Arabidopsis*, rapeseed, and cotton (Baumann et al. 2015; Kwon et al. 2020; Lee et al. 2019; McGarry et al. 2013; Sriboon et al. 2020). Previous studies have demonstrated the manipulation of petunia architecture by altering the *GAI*, resulting in plants with retarded growth (Liang et al. 2014). Finely tuned regulatory genes control the shift of plants from vegetative growth to reproductive growth; this includes the gibberellic acid pathway (Amasino and Michaels 2010; Boss et al. 2004).

To further investigate the cross-talk of the *PhTFL1* and *PhFT* mutated genes to their related genes, the expression of three related genes was selected, namely, *PhGAI*, *PhSOC*, and *PhCO* (Dill and Sun 2001). In this study, mutant lines showed an increase in the expression of *PhCO* compared to wild-type plants, which indicates a positive relation with early flowering, as previously reported in *Arabidopsis* under long-day conditions (Luccioni et al. 2019). Overexpression of *PhSOC* also regulates the flowering time under the regulation of GA and FT (Jung et al. 2012; Ruokolainen et al. 2011).

## Conclusion

In conclusion, the development of the mutant Petunia lines using the CRISPR/Cas9 technology has resulted in the production of a compact and bushy architecture with early flowering. These are highly advantageous traits in the ornamental plants industry, particularly for bedding plants both indoors and outdoors. The strategy employed facilitates rapid and efficient engineering of the Petunia genome to conform to the most challenging agronomic parameters in the ornamental plant industry, characterized by bushy and compact plant size. Through our CRISPR-Cas9-based approach, the rapid modification of numerous other ornamental plants into a more aesthetically appealing with compact growth and early flowering characteristics through the generation of loss-of-function alleles of *FT/TFL1* within elite breeding lines is possible. Alternatively, in cases where resources for genome editing are unavailable, the genetic diversity obtained within these genes could seamlessly be integrated into the traditional breeding programs in ornamental Petunia.

### Electronic supplementary material

Below is the link to the electronic supplementary material.

Supplementary Material 1

Supplementary Material 2

Supplementary Material 3

## Data Availability

All data generated or analyzed during this study are included in this published article (and its Supporting Information files). The materials used in our study are available under an MTA from the corresponding author upon reasonable request.

## Abbreviations

*ATC*: *Arabidopsis ortholog of CENTRORADIALIS*
BAP: 6-benzylaminopurine
*BFT*: *BROTHER OF FT*
CC: Co-Culture
CD: Conserved Domain
CTAB: Cetyltrimethylammonium bromide
GFF3: Generic Feature Format version 3
KO: Knock-out
MM0: number of predicted mismatches at 0 bp
PGRs: Plant growth regulators
PPI: Protein-Protein interaction
SRI: Selective root-inducing
SSI: Selective Shoot Induction
TSF: TWIN SISTER OF FT
